# Effects of a randomized controlled intervention trial on return to work and health care utilization after long-term sickness absence

**DOI:** 10.1186/s12889-016-3812-4

**Published:** 2016-11-09

**Authors:** Anne-Mette H. Momsen, Christina Malmose Stapelfeldt, Claus Vinther Nielsen, Maj Britt D. Nielsen, Birgit Aust, Reiner Rugulies, Chris Jensen

**Affiliations:** 1 DEFACTUM - Social & Health Services & Labour Market, Central Denmark Region, Aarhus, Denmark; 2 Section of Clinical Social Medicine and Rehabilitation, Institute of Public Health, Aarhus University, Aarhus, Denmark; 3 COWI A/S, Lyngby, Denmark; 4 The National Research Centre for the Working Environment, Copenhagen, Denmark; 5 Department of Public Health, University of Copenhagen, Copenhagen, Denmark; 6 Department of Psychology, University of Copenhagen, Copenhagen, Denmark; 7 National Centre for Occupational Rehabilitation, Rauland, Norway; 8 Department of Public Health and General Practice, Norwegian University of Science and Technology, Trondheim, Norway; 9 MarselisborgCentret, P.P. Oerums Gade 11, 1B, 8000 Aarhus C, Denmark

**Keywords:** Effect evaluation, Health care utilization, Interdisciplinary intervention, Randomized controlled trial, Rehabilitation, Return-to-work, Sickness absence, Somatic symptoms, Health anxiety

## Abstract

**Background:**

The aim of the RCT study was to investigate if the effect of a multidisciplinary intervention on return to work (RTW) and health care utilization differed by participants’ self-reported health status at baseline, defined by a) level of somatic symptoms, b) health anxiety and c) self-reported general health.

**Methods:**

A total of 443 individuals were randomized to the intervention (*n* = 301) or the control group (*n* = 142) and responded to a questionnaire measuring health status at baseline. Participants were followed in registries measuring RTW and health care utilization. Relative risk (RR) and odds ratio (OR) were used as measures of associations. Results were adjusted for gender, age, educational level, work ability and previous sick leave.

**Results:**

Among all responders we found no effect of the intervention on RTW. Among participants with low health anxiety, the one-year probability of RTW was lower in the intervention than in the control group (RR = 0.79 95 % CI 0.68-0.93), but for those with high health anxiety there was no difference between the groups (RR = 1.15 95 % CI 0.84-1.57). Neither general health nor somatic symptoms modified the effect of the intervention on RTW. The intervention had no effect on health care utilization.

**Conclusions:**

The multidisciplinary intervention did not facilitate RTW or decrease health care utilization compared to ordinary case management in subgroups with multiple somatic symptoms, health anxiety or low self-rated health. However, the intervention resulted in a reduced chance of RTW among participants with low health anxiety levels.

**Trial registration:**

ISRCTN43004323, and ISRCTN51445682

## Background

Because of the high human and economic costs of sickness absence and disability benefits, researchers and practitioners in many Western countries have been trying to develop interventions to facilitate return to work (RTW) [1].

Some multidisciplinary intervention studies integrating efforts in healthcare, at the work place and in disability case management for absentees with musculoskeletal disorders (MSD) have shown an effect on faster RTW as compared to treatment as usual [2–4]. A recent systematic review also found that community and workplace-based interventions in workers with MSDs were able to reduce sickness absence and job loss. However, the benefits of interventions were small and their cost-effectiveness remains uncertain [5]. The review could not identify a particular intervention that was clearly superior to others. The review further showed that high-quality studies reported smaller effects than low-quality studies. Later studies have reported no, ambiguous or even negative effects on RTW for sickness beneficiaries [6–9]. Several studies show that early interventions are preferable, however a better understanding of which specific components of the interventions work for different sickness beneficiaries is clearly needed as interventions may have different effects on different subgroups of participants [10–13].

Poulsen et al. suggested that comprehensive multidisciplinary interventions may be more appropriate for sickness beneficiaries with complex reasons for not returning to work than for beneficiaries with less complex reasons [9, 14]. Highly complex reasons may include long duration of sickness absence, problematic social relations at work, poor health and unclear diagnoses based on multiple symptom patterns [15–17].

In this randomized controlled trial (RCT) study we investigate if the effect of a multidisciplinary intervention in the Danish National RTW program [18] on RTW and use of health care differed by the participants’ self-reported health status. We focus on multiple somatic symptoms, health anxiety and poor general health, all of which may add to the complexity of the health status of sickness beneficiaries. Multiple somatic symptoms including musculoskeletal symptoms are the most common causes of sickness absence [16, 19–21]. Health anxiety and poor general health often accompany cases with an unclear diagnosis, but they are also independent risk factors for delayed RTW [15].

Patients with complex health problems and delayed RTW are also a challenge in health care, and general practitioners are frequently confronted with patients presenting multiple symptoms [22, 23]. Thus societal costs are high in terms of health care use and time lost from work [22].

We anticipate that multidisciplinary interventions may decrease visits in primary care for patients with complex health problems and maybe also affect health care utilization in the secondary health care sector.

The aim of the present article was therefore to examine whether the effect of the multidisciplinary intervention on RTW differed by self-reported health status at baseline. Furthermore, we examined whether there were effects on health care utilization.

## Methods

### Sickness absence management in Denmark

In Denmark the municipal jobcentres are responsible for paying sickness benefits and initiating occupational rehabilitation. All employed, self-employed, temporarily employed and unemployed persons with a history of previous employment are eligible for sickness benefits. In 2010, the employer paid full wage during the first 21 days of sickness absence, which was changed to 30 days January 1st, 2012. After this period employers could claim compensation for a part of the wage from the local municipality for a maximum of 52 weeks within a period of 78 weeks. Sickness benefits from employer and municipality could therefore be obtained for up to 55 weeks in total, but extensions could be granted. Medical certificates were not mandatory but could be requested by the municipality and the employer.

Sickness benefit officers conducted an assessment interview with all sickness beneficiaries by the end of the 8th week of sickness absence. Based on this assessment the officers assigned the beneficiaries into three categories: Category 1 included individuals who were likely to RTW within three months; category 2 included individuals who were unlikely to RTW within three months, but who were able to participate in activities that may facilitate RTW. Category 3 included individuals who were unlikely to RTW within three months and unable to participate in RTW activities. Only individuals in category 2 were eligible for the multidisciplinary intervention. For individuals in category 2 follow-up interviews were required with the municipal sickness benefit officer at least every fourth week.

For all beneficiaries an individual RTW plan was developed. This could include work ability training, gradual RTW, work modifications, education and exercises. Sickness benefit regulations do not specify which kind of activities should be available and the activities therefore varied between municipalities [24, 25].

### Study design

The design of the Danish National RTW program has been described earlier [18], including a thorough description of the RCT conducted in three of the municipalities [9, 14].

We performed a sub-group analysis among beneficiaries in one of these municipalities, and collected additional data on baseline health status with regard to multiple somatic symptoms, health anxiety and general health. In this municipality the overall analyses had shown no effect of the intervention on time of sickness absence [9], and time to self-support [14] and an even negative effect of the intervention on time to self-support among initially employed participants [14].

As soon as beneficiaries were assigned to category 2, they were randomized to either intervention or control group, i.e., ordinary sickness benefit management. Due to a fixed budget the allocation ratio was regularly adjusted by the National Research Centre for the Working Environment; thus the participants allocated to the RTW intervention were recruited until the target number of participants was reached.

### Participants

Eligible participants were category 2 beneficiaries between 18-65 years, who were asked to meet at the municipal jobcentre for their first interview after sickness absence. Participants were included between 1st January 2011 and 1st June 2012.

### RTW intervention

The RTW intervention was integrated in the excisting framework for sickness absence management and consisted of three core components: establishment of multidisciplinary RTW team, introduction of standardized work ability assessment procedures and tools and a comprehensive RTW training course for all team members. All participating municipalities were required to establish one multidisciplinary RTW team per 170 recruited category 2 beneficiaries annually. One team consisted of two RTW coordinators (sickness benefit officers) and health professionals (e.g., a psychologist, a physiotherapist, a psychiatrist and a physician specialised in occupational, social or general medicine). In the first interview the RTW coordinators used an extensive standardized assessment tool, including a screening questionnaire for mental health problems. Based on the assessment, the RTW coordinator decided whether or not to refer beneficiaries to other team members. The RTW team discussed these cases at weekly meetings and developed an RTW plan tailored to the needs of the benficiary. RTW coordinators could also involve the RTW team members in RTW activities, e.g., in the cooperation with general practitioners and employers. Furthermore, the psychologists and physical therapists were responsible to establish group education and training sessions e.g., on psycho-education, ergonomics training, physical exercises, stress and pain management. (For further details about the content of the intervention see Aust et al.) [18, 26].

### Control group

In ordinary sickness absence management, social benefit officers were also obliged to make a RTW plan, and the municipalities are also responsible for initiating RTW activities. However, in ordinary sickness benefit management social insurance officers do not have access to a multidisciplinary team within the municipal job center. Therefore in ordinary sickness benefit management social insurance officers do not have the possibility to discuss cases with a team of health professionals or include them directly in contacts with other physicians or employers.

### Data

Data were retrieved through questionnaires, administrative jobcentre registries and national registries. The questionnaire was mailed to the participant after randomization. If no response was received, one phone call reminder was provided after three weeks.

### Outcome variables

We retrieved data on RTW and benefits from the Danish Register for Evaluation of Marginalization (DREAM). DREAM contains information on all social transfer payments including sickness benefits paid by the state or municipality on a weekly basis [27–29].

RTW was defined as ceasing of sickness absence payments, i.e., the beginning of the first four consecutive weeks of either self-support or where unemployment benefits were received whilst the individual was applying for work (unemployment benefits cannot be paid to sickness beneficiaries).

We obtained data on health care utilization from the Danish National Patient Registry (number of contacts with own general practitioner, visits in physiotherapist clinics, and number of visits in somatic and psychiatric out-patient clinics). Number of admissions in somatic and psychiatric hospitals was obtained from the Danish Register of Hospital Utilization. For all study participants we analysed the number of contacts within the National Patient Registry and the number of hospital admissions during the year preceding baseline and during the one year follow-up period.

### Background variables

Baseline was defined by the date of response to the questionnaire. The questionnaire contained validated instruments on socio-demographic variables, work-related factors [30], employment status, and three measures of self-reported health status; multiple somatic symptoms, health anxiety and general health.

Information concerning participants’ type of work at baseline, previous employment and duration of sickness absence one year prior to baseline was identified in DREAM.

Duration of status as employed or unemployed during the year prior to baseline was measured as the total number of weeks with no social transfer payment and/or unemployment benefit. Previous sickness absence was calculated by adding the number of sickness benefit weeks in the year prior to baseline.

We assessed multiple somatic symptoms with the symptom check list (SCL-SOM) scale [31]. The questions asked about the extent the participants were bothered during the last 4 weeks by 12 symptoms: “headaches, dizziness or faintness, pains in heart or breast, pains in lower back, nausea or upset stomach, soreness of muscles, trouble getting your breath, hot or cold spells, numbness or tingling in parts of your body, a lump in the throat, feeling weak in parts of your body, heavy feelings in your arms or legs” [32].

Responses to the SCL-SOM questions were scored on a five-point Likert scale (0-4) ranging from “not at all” to “extremely” and were summed up in a severity score ranging from 0 to 48 points [23]. This method has been validated in previous studies where it showed to be comparable with a semi-structured psychiatric interview [32, 33].

We dichotomised the baseline sum score of SCL-SOM with a cut-point of <16 vs. > = 16; because this cut-point was found to be optimal for the prediction of RTW (non-published data from the same study population).

Symptoms of health anxiety were assessed using the Whiteley scale, originally derived from the Illness Behaviour Questionnaire [34] which has shown good internal and external validity [35, 36]. The Whiteley scale includes 7 questions: “worries that there is something seriously wrong with your body, worries that you suffer a disease you have read or heard about, many different pains and aches, worries about the possibility of having a serious illness, many different symptoms, thoughts that the doctor may be wrong if telling you not to worry, worries about your health” [32].

Responses were scored on the same scale as SCL-SOM ranging from 0 to 28 points.

Health anxiety was dichotomized at <7 vs. > = 7 according to the median baseline sum score (7 inter-quartile range (iqr) 3-12).

General health was based on a question from the short-form health survey (SF-36): “In general, would you say your health is excellent, very good, good, fair or poor?” [30, 37]. General health was dichotomised in fair-poor vs. good-excellent categories which have been used in another study of patients predicting RTW after sickness absence [38].

### Information retrieved from the jobcentre

Data on the participants’ self-reported reasons for sickness absence elicited through open-ended questions were retrieved from administrative forms completed by the sickness benefit officers. We categorized these causes into six groups: 1) musculoskeletal disorders (MSD), 2) common mental disorders (CMD), 3) stress, 4) cardiovascular disease/lung disease/cancer, 5) functional somatic syndromes (including muscle pain/fibromyalgia, whiplash syndrome, chronic fatigue syndrome, and unknown reasons for ill health), and 6) other (including allergies, infections, diabetes, neurological disorders, skin, eye or ear disorders, metabolic disorders, and other).

### Analysis

Non-response analyses were performed on demographic variables, reason for sickness absence, and health care utilization, duration of sickness absence and employment status one year prior to baseline, as well as labour market participation one year after the intervention. Descriptive analyses of background variables, self-reported reason for sickness absence, work-related factors and duration of previous sickness absence were carried out. Chi-square or Fisher’s exact test were performed for categorical variables, independent Student t-tests and Wilcoxon rank sum test were performed for continuous data.

We analysed whether multiple somatic symptoms, health anxiety or general health modified the effect of the intervention on RTW and performed stratified analyses with the same variables.

The relative risk (RR) of experiencing RTW within 52 weeks was analyzed in a generalised linear regression model. The pseudo values method [39] was used to be able to take competing risks (old age pension, disability pension or death) and individual follow-up durations until RTW occurred into account. Hence, the cumulative incidence proportion (CIP) as a function of the number of follow-up weeks was estimated using the Kaplan-Meier curve.

We chose the pseudo values method instead of the more commonly used Cox proportional hazard regression, because the event RTW was more incident than 10 %, and therefore the hazard ratio and RR would not be equivalent. Furthermore, the pseudo values method allows for censoring due to competing risks and thereby individual time at risk before RTW is accounted for like in Cox regression [39].

The outcome consisted of two measures: did RTW occur (yes or no) and the time until the event was identified in DREAM, end of follow-up or competing risks occurred, whichever came first.

In the analyses of whether multiple somatic symptoms, health anxiety or general health modified the effect of the intervention on RTW, we adjusted for the following potential confounders: gender, age, education level, work ability, and sickness absence during the previous year.

To explore possible differences of employed vs. unemployed beneficiaries’ RTW process, sensitivity analyses were carried out restricted to participants with at least 13 weeks of employment during the previous year [40].

Analyses of health care utilization one year after the RTW intervention were carried out for all participants and additionally for participants with high scores of somatic symptoms and health anxiety and low score in general health. Median number of health care provider visits and the corresponding interquartile range (iqr) were summarized. Additionally, as in a previous study, the number of visits in general practice was dichotomized (0-6 /> = 7) [9]. Finally, the remaining visits in (physiotherapists’ clinic, out-patients’ clinic, and the number of admissions in hospitals) were also dichotomised (0/> = 1) [14].

It was analysed whether multiple somatic or health anxiety symptoms, or general health modified the effect of the intervention on use of health care services one year after the RTW intervention in logistic regression analyses. Effects were adjusted for gender, age, and education-level.

## Results

**Fig. 1:**
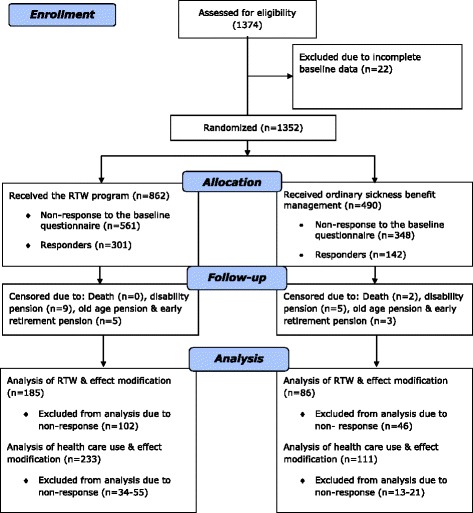
Flow chart of the recruitment procedure and the drop-outs during follow-up

In total, 1352 participants were randomised to the RTW intervention (*n* = 862) or sickness management as usual (*n* = 490, Fig. 1). The response rates to the questionnaire were 35 % in the RTW intervention group and 29 % in the control group.

Table 1 shows baseline characteristics of responders and non-responders. There were no statistically significant differences regarding age, gender, type of work, or use of most health care services (general practice, physiotherapists, outpatient clinics, psychiatric outpatient clinics, admissions in somatic hospitals). However, the responders had significantly fewer admissions to psychiatric hospitals: the median number of admissions was 5 among responders vs. 20 among non-responders. Responders also reported significantly more often MSDs and less often CMDs as reason for sickness absence.

**Table 1:** Baseline characteristics of the non-responders and responders

*N* = 1352	Non-responders *n* = 909	Responders *n* = 443	*p* value
Women, *n* (%)	520 (57)	273 (62)	0.12 A
Age, average years (SD)	41.3 (11.8)	44.4 (10.8)	0.05 B
Work *n* (%)			0.15 A
Farming, fishery, industry	150 (17)	65 (15)	
Construction, trading and transport	231 (25)	109 (25)	
Communication, information, finance, insurance, real estate and consultancy	103 (11)	49 (11)	
Public administration, teaching, health, culture and leisure	329 (36)	189 (43)	
Other	36 (4)	11 (2)	
Missing	60 (7)	20 (5)	
Self-reported reason for sickness absence *n* (%)			<0.001 A
Musculoskeletal disorder (MSD)	387 (43)	244 (55)	
Common mental disorder (CMD)	294 (32)	94 (21)	
Stress	95 (19)	52 (12)	
Functional somatic syndrome/unknown	19 (2)	10 (2)	
Heart disorder, lung disorder, cancer	36 (4)	12 (3)	
Other	78 (9)	31 (7)	
*Previous year before inclusion* median (iqr)			
Employment or unemployment, weeks	42 (26-46)	42 (33-45)	0.35 C
Duration of sickness absence, weeks	7 (5-12)	7 (6-11)	0.43 C
Health care utilization			
Visits in general practice	11 (7-18)	12 (7-18)	0.30 C
Visits in physiotherapist clinics	0 (0-0)	0 (0-1)	0.30 C
Visits in out-patient clinics	2 (1-6)	3 (1-6)	0.58 C
Visits in psychiatric out-patient clinics	0 (0-0)	0 (0-0)	0.19 C
Admissions in hospitals	0 (0-0)	0 (0-0)	0.45 C
Admissions in psychiatric hospitals	0 (0-0)	0 (0-0)	0.02 C
*One year after inclusion*			
Labour market participation *n* (%)			0.92 A
Non-RTW	309 (34)	146 (33)	
RTW	548 (60)	273 (62)	
Old age and disability pension	50 (6)	22 (5)	
Died	2 (0)	2 (0)	

A Chi2 test, B *t*-test, C Wilcoxon rank sum test

No significant difference was found between responders and non-responders regarding labour market participation.

Table 2 shows baseline characteristics for the participants of the intervention group and the control group. Demographic factors (age, gender, education level) general health, score of health anxiety symptoms, reason for sickness absence, work ability and work-related factors (support from supervisor and co-workers, and fear of losing job) were similar in the two groups.

**Table 2:** Baseline characteristics of study groups (intervention compared to control)

[n for whom data available]	Intervention group (*n* = 301)	Control group (*n* = 142)	*p* values
Demographic variables			
Women, *n* (%) [273]	179 (59)	94 (66)	0.17 A
Age, average years (SD) [443]	44.2 (11.2)	44.6 (10.2)	0.74 B
Education level, *n* (%) [305]			0.35
No education or under education	36 (14)	17 (14)	
Skilled worker	73 (29)	41 (34)	
Basic or middle level	159 (55)	36 (47)	
High level (>4 years)	5 (2)	5 (4)	
Health factors			
Self-rated health, good-excellent *n* (%) [373]	143 (56)	59 (50)	0.33 A
SCL-SOM sum score > =16, *n* (%) [414]	125 (45)	70 (52)	0.15 C
SCL-SOM sum score, median (25 %, 75 %) [414]	14 (0, 36)	16 (2, 40)	0.02 C
Whitely sum score > =7, *n* (%) [415]	132 (47)	70 (52)	0.32 C
Whitely sum score, median (25 %, 75 %) [415]	7 (0, 25)	8 (0, 4)	0.24 C
Self-reported reason of sickness absence, *n* (%) [443]			0.53 A
Musculoskeletal disorder (MSD)	170 (56)	74 (52)	
Common mental disorder (CMD)	56 (19)	38 (27)	
Stress	38 (13)	14 (10)	
Functional somatic syndrome/unknown	7 (2)	3 (2)	
Heart disorder, lung disorder, cancer	8 (3)	4 (3)	
Other	22 (7)	9 (6)	
Employment status, *n* (%) [311]			0.36 D
Employed at inclusion	175 (81)	84 (88)	
Laid off	36 (17)	10 (10)	
Left job	4 (2)	2 (2)	
Work ability *n* (%) [375]			0.23 A
Poor (0-1)	86 (33)	49 (42)	
Moderate (2-4)	109 (42)	40 (34)	
Excellent (>4-10)	62 (24)	29 (25)	
Perceived workability in 2 years, yes [268]	180 (73)	88 (74)	0.83 A
Work-related factors, *n* (%)			
Support by supervisor, yes [232]	159 (94)	73 (94)	1.00 D
Support by co-workers, yes [241]	166 (98)	75 (95)	0.27 D
Afraid of losing job, yes [95]	66 (40)	29 (37)	0.65 A
*Previous year before inclusion*, median (25 %,50 %) [443]			
Employment or unemployment, weeks	43 (0, 50)	41 (0, 48)	0.20 C
Duration of sickness absence, weeks	7 (1, 29)	7 (2, 27)	0.15 C
Health care utilization			
Visits in general practice	11 (1, 37)	14 (1, 33)	0.007 C
Visits in physiotherapist clinics	0 (0, 45)	0 (0, 17)	0.99 C
Visits in out-patient clinics	3 (0, 19)	3 (0, 20)	0.69 C
Visits in psychiatric out-patient clinics	0 (0, 17)	0 (0, 11)	0.08 C
Admissions in hospitals	0 (0, 3)	0 (0, 2)	0.49 C
Admissions in psychiatric hospitals	0 (0, 0)	0 (0, 0)	0.58 C

A Chi2 test, B *t*-test, C Wilcoxon rank sum test, D Fischer's exact test

The median duration of previous sickness absence was 7 weeks at inclusion in both groups. During the year before inclusion the intervention group had less visits in general practice compared to the control group (11 vs. 14). The intervention group also scored lower in the SCL-SOM sum score than the control group (median 14 vs. 16, results not shown).

### Effect of RTW intervention

The total time at risk was 13,259 weeks before RTW (*n* = 265), competing risk (*n* = 21) or censoring (*n* = 146) occurred. At time zero, eight absentees returned to work and three experienced one of the competing risks and were subsequently excluded from the final analyses.

Among all participants there was no effect of the intervention on RTW (RR 0.92, 95 % CI 0.78-1.08, Table 3). There was also no intervention effect when we stratified the analyses by high vs. low multiple somatic symptoms. Those with good general health had a significantly reduced chance of RTW (RR 0.84, 95 % CI 0.74-0.97) in the intervention group, but high and low general health did not significantly modify the effect of the intervention on RTW (*p* = 0.18). However, stratifying by high vs. low health anxiety showed a significant interaction effect (*p* = 0.04). Among participants with low health anxiety, the chance of RTW was lower in the intervention group than in the control group (RR = 0.79 95 % CI 0.68-0.93), whereas there was no effect of the intervention among high health anxiety participants (RR = 1.15 95 % CI 0.84-1.57).

**Table 3:** Return to work (RTW) in intervention and control group for all participants and for subgroups with different health status at baseline analysed by the pseudo values method

	Intervention group (IG) (*n* = 185)	Control group (CG) (*n* = 86)	RTW for IG with CG as reference group	Inter-action between groups and health status on RTW
	Median weeks until RTW (iqr)		RR (95 % CI)^a^	*p* value
All participants [271]	30 (13-51)	23.5 (10-51)	0.92 (0.78-1.08)	
Somatic symptoms				
High > =16 [195]	46 (16-51) *n* = 125	33.5 (15-51) *n* = 70	0.82 (0.63-1.08)	0.36
Low [219]	24 (11-51) *n* = 155	15 (9-41.5) *n* = 64	0.96 (0.79-1.16)	
Health anxiety				
High > =7 [202]	37.5 (16.5-51) *n* = 132	42 (14-51) *n* = 70	1.15 (0.84-1.57)	0.04
Low [213]	25 (11-51) *n* = 149	15.5 (8.5-27) *n* = 64	0.79 (0.68-0.93)	
General health				
Poor [171]	46 (19-51) *n* = 113	48 (16-51) *n* = 58	1.13 (0.76-1.67)	0.18
Good [202]	24 (11-49) *n* = 143	15 (8-30) *n* = 59	0.84 (0.74-0.97)	

^a^Adjusted for: gender, age, education level, work ability, sickness absence previous year

When we repeated the analyses for participant with at least 13 weeks of employment prior to inclusion to explore whether duration of employment affected the results, we found similar results (results not shown).

### Health care utilization

There was no effect of the intervention on any measure of health care utilization during follow-up. In the intervention group 74 % of the beneficiaries visited general practice > =7 times during follow-up vs. 79 % among the control group. The median number of visits were 11 and 14, respectively (OR 0.9, 95 % CI 0.53-1.55). There was also no significant interaction effect of health status on the intervention for visits in general practice after adjusting for gender, age and educational level. For participants with high levels of multiple somatic symptoms the median number of visits to general practitioners was 14 in the intervention group and 15 in the control group. For participants with high anxiety levels the median numbers of visits to general practitioners were 14 and 15 and for those with poor general health the median number of visits were 15 and 16 in the intervention and control groups, respectively.

## Discussion

We did not find an overall effect of the RTW program on RTW and health care utilization.

However, we found that the intervention compared to ordinary sickness management resulted in a reduced chance of RTW among participants with low health anxiety levels. Effect modification was not present for somatic symptoms or general health.

The evaluation of the Danish RTW program did not find an effect on duration of receiving sickness absence benefits and time to self-support in two of three municipalities where an RCT design was used [9, 14]. The authors discussed whether the multidisciplinary RTW intervention might be more effective in cases with a history of longer sickness absence indicating more complexity [9]. The results of our study did not support the idea that this specific multidisciplinary intervention might be more effective for beneficiaries with more complex health status [41]. However, the intervention seemed to reduce the chance of RTW for participants with low health anxiety and maybe also among those with good general health. An explanation might be that these cases were “over-treated” resulting in prolonged sickness absence [15].

Barriers for RTW may include factors related to both physical and psychological aspects in a complex interplay with the possibilities of the work place to accommodate employees with reduced work ability. While somatic symptoms often are examined early in the sickness absence period and diagnosed if possible, the work ability of the person also depends on the demands at the work place and the person’s beliefs in her- or himself to meet these demands. Brouwers et al. suggested that RTW programmes may yield better results on both RTW and reduced use of health care if targeted at a sub-group of beneficiaries with more severe problems or if carried out closer to the workplace [41]. Kuoppala showed in a systematic review that workplace integration in rehabilitation is essential [42]. The national RTW program aimed at improving communication with the employers of the beneficiaries and workplace integration. However, among beneficiaries who were employed when their sickness absence started only 9 % had at least one meeting with their workplace [26]. Thus, work place participation in the present intervention programme was not achieved to any significant extent. As the effects of the intervention were modified by levels of health anxiety, it is possible that RTW would have been faster for a subgroup of beneficiaries if work place participation had been implemented.

The intervention aimed to facilitate RTW primarily by including health professionals in the assessment of sickness beneficiaries at the job centres. This may partly explain why we found no intervention effect on health care utilization, neither in those with many symptoms nor in those with fewer symptoms. RTW intervention studies are often carried out in clinical practice, such as in occupational health care or hospital settings, whereas the present study was carried out in the municipal job centre. Although the health professionals in the RTW intervention were not allowed to provide treatment, they were supposed to advise the beneficiaries on health issues and RTW strategies. This may have been a reason for the lack of effect on health care utilization in the intervention group.

### Strengths and limitations

The main strengths of the study are the RCT design and the use of data from national registries to measure outcomes. It is a strength that baseline characteristics were not statistically significant different between the groups, i.e., demographics, employment status; self reported health, and work ability.

The main weakness of the study is the low response rate. It is unknown to us whether responders have been more likely to participate in activities or had a more favourable attitude towards study components than non-responders. However, our endpoint, i.e., labour market participation, was not statistically significant different between responders and non-responders.

Despite several attempts, it was not possible to collect reliable data about each beneficiaries’ use of the different intervention components, which is a limitation [26]. The knowledge of treatment status may have influenced the difference in response rate, as participants receiving the RTW intervention may have had a more favourable attitude towards responding and more positive expectation. However, it is not possible to blind participants in this type of intervention studies.

Another limitation of the study is that the reasons for sickness absence are self-reported, and therefore it was not verified if the symptoms were non-specific. Responders reported significantly more often MSDs and less often CMDs or stress as the reason. There were also fewer admissions to psychiatric hospitals among responders. CMDs or stress may negatively influence the response rate in questionnaires for participants. This self-selection of participants with regard to better mental health and thereby higher chance of RTW, may limit the external validity.

One third of the beneficiaries were unemployed at the time of inclusion and their chance of resuming work was lower than for employed beneficiaries. We defined RTW as having ceased to receive sickness benefits. Some returned to work, whereas others were fit to work and received unemployment benefits, but had no work to return to. However, the sensitivity analyses indicated that the findings were robust, i.e., the differences between the groups were similar when only employed participants were analysed.

Participants were categorized into category 2, and herewith became eligible for our study, not based on medical criteria, but based on the sickness benefit officer’s’ assessment that the person was unlikely to RTW within three months but was able to participate in RTW-facilitating activities. That selection of participants was based on neither medical criteria nor self-rated health but on administrative practices to assess and predict future work ability. This has both advantages and disadvantages. An advantage is that we used the same selection criterion, likelihood for RTW and ability to participate in RTW-facilitating activities, which are used in all Danish job centres and may have ensured ecological validity of our study. However, category two has shown to not be as reliable as assumed because the criteria for selection are used differently [26]. Another disadvantage is that we lack information on the seriousness of the underlying health problem. However, to improve practice in management of beneficiaries, we believe that it is important to adhere to criteria that are used in the job centre and not rely on criteria that are used in the health care system. Work ability assessments in the municipal job centre system partly rely on health assessments, but also depend on other factors such as personal and environmental factors, and work ability therefore cannot be deduced from diagnostic assessments alone.

## Conclusions

The multidisciplinary intervention did not facilitate RTW more than ordinary sickness management in subgroups with multiple somatic symptoms, health anxiety or low self-rated health. However, the intervention resulted in a reduced chance of RTW among participants with low health anxiety levels, but more research including work place integration in RTW programs is needed.
